# Hyperprogressive Disease in Malignant Carcinoma With Immune Checkpoint Inhibitor Use: A Review

**DOI:** 10.3389/fnut.2022.810472

**Published:** 2022-03-25

**Authors:** Xiaojun Liu, Liang Qiao

**Affiliations:** ^1^The Second Department of Radiotherapy, Gansu Provincial Hospital, Lanzhou, China; ^2^The First Clinical College, Chinese Medical University of Gansu, Lanzhou, China; ^3^The First Clinical College, Lanzhou University, Lanzhou, China; ^4^Storr Liver Centre, Westmead Millennium Institute for Medical Research, The University of Sydney, Westmead, NSW, Australia

**Keywords:** cancer, immune checkpoint inhibitors, hyperprogressive disease, outcome, immunotherapy

## Abstract

Immune checkpoint inhibitors (ICIs) have shown durable remissions and improved long-term survival across a variety of cancer types. However, there is growing evidence that a significant subset of nonresponsive patients may exhibit hyperprogressive disease (HPD) during the initiation of immune checkpoint inhibitors (ICIs). Moreover, patients with HPD triggered by ICIs are always correlated with a deteriorating quality of life and poor prognosis. The ability to predict such rapid disease progression phenotypes is of great importance. More precision parameters to evaluate the response pattern to ICIs are urgently needed. To date, the mechanisms of HPD are still unclear. Aberrant alterations of driven genes, tumor microenvironment, or T cell immunophenotype may involve in HPD. In this article, we aim to provide an updated overview of available studies on HPD and summarize the potential predictors associated with HPD and the underlying mechanisms of HPD.

## Introduction

Immunotherapy by immune checkpoint inhibitors (ICIs) has demonstrated therapeutic responses across a variety of cancer types. However, the response patterns elicited by ICIs have been observed to differ from that of conventional cytotoxic and targeted therapy, which include relative long-term benefit, delayed response, and pseudoprogression. There is growing evidence that a significant subset of nonresponsive patients may experience an aggressive disease progression early after ICIs, a phenomenon known as hyperprogressive disease (HPD). Moreover, HPD is usually occurred along with the severe deterioration of a patient's performance status and dismal prognosis. Therefore, the ability to predict such rapid disease progression phenotypes is of great importance to protecting patients from the harmful effects of HPD. However, the definition of HPD has not yet been clearly established, and no molecular markers that could predict it have been identified. In this article, we aim to summarize updated, clinically-oriented data of available studies and explore the possible mechanisms of HPD.

## Definition of HPD

Recently, great concern has been raised over HPD caused by ICIs, and a series of studies, thus, analyzed such a novel aggressive pattern of ICIs. HPD was first described by Champiat et al. (1), based on one of two parameters that include tumor growth rate (TGR) and tumor growth kinetics (TGK). TGR assumes an exponential growth in the tumors and calculates tumor volume based on diameter. Therefore, it is defined as the log-scale calibrated change in the sum of the longest diameters (SLDs) of the target lesions according to Response Evaluation Criteria in Solid Tumors 1.1 (RECIST 1.1) per month. Similarly, TGK is defined as the change in the sum of the SLD of the target lesions according to RECIST 1.1 per month, without a log-scale calibration. Accordingly, HPD refers to progress at the first evaluation and a 2-fold or greater increase in TGR or TGK during ICIs in comparison with pretreatment duration (2). At present, TGR ≧2 is the most widespread used method evaluating HPD treated with ICIs.

However, some HPD definitions were not based on TGR or TGK criteria. A study defined HPD as a time-to-treatment failure (TTF) <2 months or > 50% increase in tumor burden compared to preimmunotherapy imaging and > 2-fold increase in progression pace (2). Another report (3) defined HPD as several methods, including progression at first restaging on ICI, or increase in tumor size > 50%, or > 2-fold increase in TGR. Besides TGR and TGK, TTF shorter than 2 months is considered an alternative assessment method for HPD. Other indices, such as tumor size, PD at first evaluation (4), fast progression (5), or even early death, were used (6). There is still no consensus on how to evaluate this phenomenon.

A more reliable definition is that HPD should have the ability to identify as many patients as possible with much poorer survival. In a gastrointestinal cancer study, seven definitions of HPD were compared and it was found that TGK served as a more convenient method to reflect tumor growth acceleration compared to TGR. In addition, incorporating new lesions emerging during the treatment was shown to be reliable for the evaluation of TGK.

Undoubtedly, the use of different definitions of HPD brings the risk of describing different tumoral behaviors. Some definitions of HPD were not associated with the same tumor behavior (7). The different definitions may result in a wide variation in HPD incidences. ICI-induced HPD remains controversial because of a lack of consensual definition, and HPD definitions are very heterogeneous in terms of incidence rate and clinical impact (8–10).

## HPD Specificity to ICI Treatment

Hyperprogressive disease may occur during the natural course, chemotherapy, or tyrosine kinase inhibitor period in patients with cancer. By using the TGR ratio method, a study compared the HPD prevalence between ICIs and chemotherapy in patients with non-small cell lung cancer (NSCLC). HPD was observed in 13.8% (56 of 406) of patients treated with ICIs compared with 5.1% (3 of 59) of patients treated with chemotherapy (11). Another study in HCC also reproduced such a conclusion. Based on the TGR ratio method, HPD was observed in 14.5% (10 of 69) of patients treated with ICIs compared with 2.9% (1 of 69) of patients treated with tyrosine kinase inhibitors in HCC (12). These studies supported HPD a disease progression pattern highly specific to the ICI treatment.

## Distinguishing Between HPD and Pseudoprogression

Besides HPD, there is another atypical response pattern related to ICIs termed pseudoprogression, which is when the tumor size transiently increases before subsequent reduction during ICI treatment. In pseudoprogression, the initial enlargement in lesion results likely from inflammatory cell infiltration instead of tumor cell proliferation. As both pseudoprogression and real progression including HPD present with an increase in tumor size, it is vital to differentiate between the two atypical response patterns. To date, a great challenge lies in precisely evaluating the clinical efficacy of ICIs for clinicians, who aim at avoiding either premature cessation of ICIs or overtreatment with ICIs.

First, pseudoprogression lesions may be considered prematurely as PD even HPD using WHO or RECIST criteria and may, therefore, make inappropriate clinical decisions. Both RECIST and WHO criteria could underestimate the clinical benefit of ICIs in a proportion of patients. The immune-related response criteria (irRC) are a more preferable evaluation criterion for the patient receiving ICIs, in which, first documented PD is required to reconfirm after at least a 4-week interval. The superiority of the irRC criterion over conventional RECIST was verified in patients receiving ICIs in a study (13). In addition, although pseudoprogression is accompanied by the initial increase of tumor size, it often follows an improved performance status (14). Conversely, HPD is always accompanied by worsened symptoms and decreased quality of life. Finally, radiological evidence alone is insufficient for clinicians to distinguish pseudoprogression from true progression or HPD when they first emerge. The best way to distinguish them is undoubtedly to conduct a biopsy of the suspicious lesions for pathologic review (15).

## HPD Incidence in Cancer Types and Clinical Significance

### HPD in Pan-Cancer Types

As shown in Table 1, in the study by Champiat et al. (1), 12 of 131 (9%) patients with cancer treated with anti-PD-1/PD-L1 experienced the HPD phenomenon. The median OS was 4.6 months in patients with HPD vs. 7.6 months in patients without HPD, though this result was not statistically significant (*p* = 0.19). The Cox regression analysis further showed that HPD was associated with higher age (> 65 years), a lower response rate, and worse overall survival but not associated with higher tumor burden or any specific tumor type.

**Table 1:** Representative case series studies of HPD in multiple cancer types.

**Reference**	**Cancer types**	**Incidence, *n* (%)**	**Clinical relevance**
Champiat et al. (1)	Pan-cancer patients	12/131 (9%)	HPD was associated with a higher age (> 65 year), poor prognosis, but not associated with higher tumor burden or any specific tumor type
Ferrara et al. (11)	NSCLC	56/406 (13.8%)	HPD was associated with high metastatic burden and poor prognosis
Zhang et al. (12)	HCC	10/69 (14.5%)	Identified three HPD risk factors including hemoglobin level, portal vein tumor thrombus, and Child-Pugh score in HCC
Saâda-Bouzid et al. (16)	Head and neck squamous cell cancer	10/34 (29%)	HPD was associated with a regional recurrence, a shorter progression-free survival.
Giuseppe et al. (17)	NSCLC	39/187 (25.7%)	Enrichment by tumor-associated macrophages within baseline cancer tissue potentially able to predict HPD
Kanazu et al. (18)	NSCLC	5 of 87 (5.7%)	HPD was associated with poor quality of life and survival
Kim et al. (19)	NSCLC	54/263 (20.5%)	HPD was associated with worse prognosis
Chen et al. (20)	Colorectal cancer	5/22 (22.7%)	HPD was associated with *KRAS* mutation

*NSCLC, non-small cell lung cancer; HCC, hepatocellular carcinoma*.

A study reviewed patients with several solid tumors who were enrolled in early-phase immunotherapy studies. The results showed that HPD occurred in 12 of 182 patients (7%). In another case series study, six of 155 (3.9%) patients with different types of cancer had a TTF <2 months after ICI treatment (2).

### HPD in HCC

Wong et al. (21) presented a case series of six patients who had demonstrated HPD while undergoing ICIs at the National Cancer Center of Singapore. In their study, the prebaseline target lesions, baseline target lesions, and the first evaluation scan were compared to assess tumor growth during the pretreatment period and treatment period. A total of 23 patients experienced rapid radiological and/or clinical progression within at least the first two cycles of ICI therapy, of which, six patients demonstrated HPD, which accounted for approximately 9% of the whole treated. All six patients were men and had cirrhosis, caused by either NASH or chronic HBV. Among them, three patients received nivolumab, one patient tremelimumab, and one patient tremelimumab combined with durvalumab. Interestingly, four of the six patients received the previous radiotherapy in the form of transarterial radioembolization with yttrium-90, which is a possible risk factor of HPD as described by Saada-Bouzid (16). Moreover, all six patients had no tumor shrinkage on a confirmatory CT scan, which excluded delayed remission and hence pseudoprogression.

In another study in HCC, HPD was observed in 14.5% (10 of 69) of patients treated with ICIs (12). Patients with HPD had a significantly shorter OS than that of the patients with non-HPD in HCC. However, there was no significant difference in OS between PD patients with and without HPD in HCC (12). In addition, three risk factors that include hemoglobin level, portal vein tumor thrombus, and Child–Pugh score were associated with HPD in HCC (12).

### HPD in NSCLC

Although ICIs have changed the treatment paradigm for patients with NSCLC, an in-depth examination of the survival curves from the CheckMate-026 (22), CheckMate-057 (23), CheckMate-227 (24), and KEYNOTE-042 (25) demonstrated an excess of disease progression and death in the immunotherapy treatment arms compared with chemotherapy in the first 3 months of treatment. In Russo et al.'s study (17), the HPD rate even reached 25.7% (39/187), and the median OS significantly decreased to 4.4 months in HPDs as compared to 17.7 in non-HPDs.

In a case series study reported by Kanazu et al. (18), five of 87 patients (5.7%) experienced HPD after the first cycle of immunotherapy. Another study assessed HPD in patients with NSCLC who received ICIs in Korea (19). Of 263 patients, HPD was observed in 55 (20.9%), 54 (20.5%), and 98 (37.3%), respectively, according to the TGK, TGR, and TTF. Patients with HPD who satisfy both TGK and TGR criteria were related to a worse prognosis. No clear relevance between clinicopathologic variables and HPD was further observed in this study (19). This is the first study to document the incidence of HPD in patients with NSCLC among the Asian population.

### HPD in Gastrointestinal Cancer

Ogata et al. (26) reported a patient with gastric cancer who received nivolumab after radiotherapy only to experience rapid progression within the irradiation field after one cycle of nivolumab therapy. This suggests that the administration of nivolumab after radiotherapy may be a risk factor for HPD in gastric cancer. To date, the mechanism of HPD within the irradiation field is not clear. Radiotherapy may lead to the release of tumor antigens or impair the immune cells in the tumor microenvironment that would change the immune environment and facilitate rapid progression within the irradiation field.

Pembrolizumab, a PD-1 monoclonal antibody (mAb), has been recommended to treat patients with metastatic deficient mismatch repair (dMMR) colorectal cancer (27). An *in vivo* study observed accelerated tumor growth after ICI treatment in mice model bearing p53-null and d-MMR colon cancer cells. This implicated p53-deficiency as a possible contributor to HPD in d-MMR tumors (28). Another retrospective clinical study analyzed HPD in pan-cancer patients and found that HPD occurred in 22.7% (5/22) of patients with colorectal cancer (20). Moreover, *KRAS* mutation was closely associated with HPD occurring in patients with colorectal cancer (20).

### HPD in Head and Neck Squamous Cell Carcinoma

In the study by Saâda-Bouzid et al. (16), HPD was observed in 10 of 34 patients (29%) with advanced head and neck squamous cell carcinoma (HNSCC) treated with ICIs. Furthermore, HPD significantly correlated with a regional recurrence, a shorter progression-free survival (PFS) but not with overall survival (OS). Otherwise, no pseudoprogressions were described in this study.

### HPD in Melanoma

Immune checkpoint inhibitors targeting PD-1 and PD-L1 have shown a significant and long-term response, either in frontline therapy or in subsequent therapies (29). Faure et al. (30) reported a case of anorectal malignant melanoma in an elderly patient treated by pembrolizumab, experiencing HPD over 2 months of treatment, then leading to rapid death.

### HPD in Renal Cancer

Renal cancer has been historically considered an immunogenic tumor. Nivolumab was approved by the FDA for patients with clear-cell renal cancer previously treated with antiangiogenic therapy based on the results from the phase III CheckMate 025 study (31). Yuki et al. (32) reported three cases of HPD during the initial phase of nivolumab treatment for metastatic renal cell cancer in the late-line setting. Clinicians should be aware of the possibility of HPD during the initial phase of nivolumab therapy, which may result in discontinuation of treatment, especially in the late-line setting of renal cancer. Additional patients need to be examined to reanalyze the possible predictive factors.

## Clinical Index and Biomarkers For HPD

The ability to predict treatment response to ICIs is, therefore, of critical importance in protecting patients from the deleterious effects of HPD. In the study by Champiat et al. (1), age appeared to be significantly associated with HPD. However, Saada-Bouzid et al. (16) and another study by Kato et al. (2) did not confirm age to be a statistically significant indicator.

Radiotherapy is considered a double-edged sword in antitumor immunity. The relation between HPD and radiotherapy is still unclear. A study by Saada-Bouzid et al. (16) found that HPD might be related to the previous radiotherapy since nine of the ten patients with HPD had recurrence in an irradiated field, which was consistent with the other three case studies (26, 33, 34). However, there are not any signs of HPD in the PACIFIC trial, where all patients were irradiated before durvalumab or placebo in Stage III NSCLC (35).

Genomic profiles may also help to identify patients at risk for HPD after immunotherapy. In a genomic prediction study (*n* = 155), four experienced HPD among the six patients with murine double minute homolog 2 (MDM2) amplification, whereas eight had a TTF <2 months and 2 developed HPD among the 10 patients with endothelial growth factor receptor (EGFR) mutations (2). This study suggests that patients for whom ICIs are planned may need genomic tests to determine whether their tumors have specific alterations correlated with HPD.

In an exploratory biomarker study, a higher ratio of severely exhausted phenotypes and a lower ratio of effector–memory subsets were correlated with HPD and shorter survival (19). Typing of CD8+ T cells before receiving ICIs may have clinical significance to predict the occurrence of HPD (19). In Russo et al.'s study (17), patients with HPD with NSCLC demonstrated tumor infiltration by M2 macrophages. Thus, given immunophenotype of T cell or macrophages are potentially able to predict HPD. Biomarkers for HPD prediction were also studied in NSCLC, which include neutrophil-to-lymphocyte ratio, lactate dehydrogenase level, and concurrence of STK11 and KRAS mutations (36).

Monitoring T cell dynamics may allow for the early detection of HPD in clinical practice and complement radiological evaluation. For example, the expression of CD28 in T cells was frequently used as a maker of differentiation degree, and expansion of CD28-CD4+ T cells in peripheral blood within the first cycle of therapy was considered an early sign of HPD in NSCLC treated with ICIs (37). Such a conclusion needs to be further confirmed in future studies.

Tunali et al. (38) established a model combining some clinicopathological covariates with radiographic image features to predict HPD in patients with NSCLC. The pretreatment baseline predictors that were used to identify these phenotypes included a series of clinicopathological data, hematological data, and radiographic features. Using this model, patients who experienced HPD or TTP <2 months were predicted with 73.4 and 82.3% of accuracy. Further studies will be required to validate the clinical utility of this model.

## Mechanisms Underlying HPD

**Figure 1:**
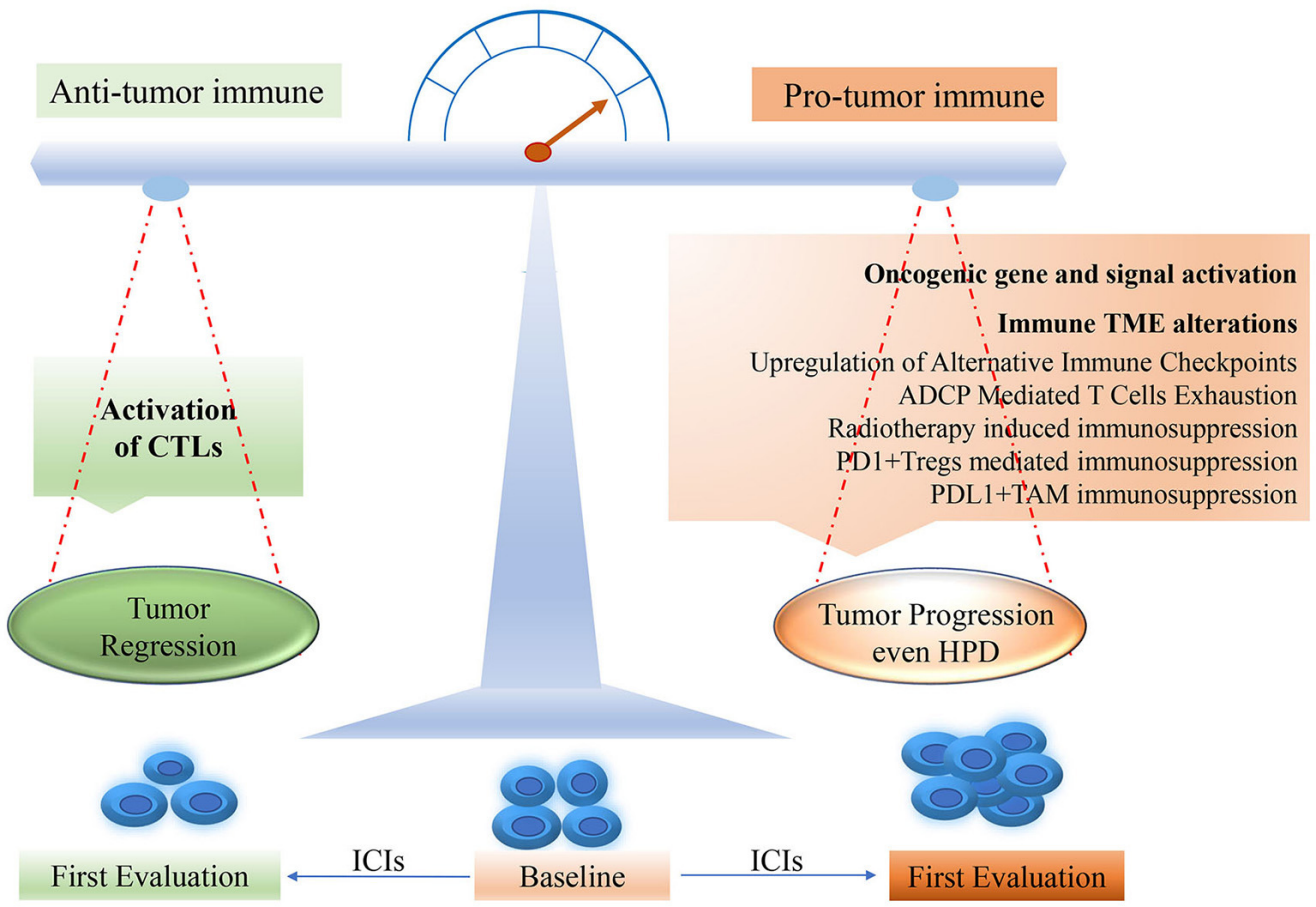
The potential mechanism for HPD occurring in ICI treatment. HPD, hyperprogressive disease; PD-1, programmed death-1; PD-L1, programmed death ligand-1; CTL, cytotoxic T lymphocyte; TME, tumor microenvironment; ICI, immune checkpoint inhibitor.

The potential mechanisms underlying HPD remain elusive, though several hypotheses have been proposed in the area of ICIs. Some findings may be helpful to reveal the mechanisms of the development of HPD after ICI treatment. As shown in Figure 1, the oncogenic signaling activation such as overexpression of MDM2 and EGFR could be triggering the acceleration of tumor growth. In addition, the changes in the tumor immune microenvironment (TME) after ICIs might contribute to HPD. Particularly, the aberrant alterations of immune cell subpopulations such as macrophages and T regulatory cells (Tregs) may account for HPD occurrence.

### Activation of Oncogenic Genes

A study analyzed the somatic alterations with next generation sequencing of tumor tissue in HPDs treatment with ICIs in advanced solid tumors and showed that copy number alterations in MDM2/MDM4, EGFR, and several genes located on 11*q*13 were associated with HPD (3). In addition, mutation patterns were altered in HPD tumors subsequent to ICIs. In posttherapy HPD tumors, several somatic mutations were observed in known cancer genes that include tumor suppressor genes such as TSC2 and VHL, along with transcriptional upregulation of cancer signaling pathways, which include IGF-1, ERK/MAPK, and PI3K/AKT (3).

Interferon-γ (IFN-γ)-driven growth due to PD-1 inhibition might be accounted for HPD. Peng et al.'s study (39) found that PD-1 blockade could increase the production of IFN-γ at the tumor site and cause rapid disease progression by MDM2/P53 interaction. Research has shown that MDM2 protein can control the activity of *P53* by blocking the *P53* transactivation domain and tagging *P53* for ubiquitin-mediated proteasome degradation (40), whereas IFN-γ can elevate the expression of MDM2 and consequently inhibit the activity of P53. In murine model research, PD-1 knockout mice infected with tuberculosis produced superabundant IFN-γ and consequently lead to fulminant disease and then sacrificed (41). However, the increasing IFN-γ production after ICIs administration is unclear yet in the clinical setting. Further studies will be needed to confirm the possible role of IFN-γ/MDM2/P53 pathways for HPD.

A study found genomic variates in SMARCA2 gene or APC signaling pathway activation might be associated with the occurrence of HPD in gastrointestinal cancer, whereas MSH6 gene or Wnt signaling pathway alterations might have a lower risk of HPD (42).

### Radiotherapy Induced a Detrimental Effect Upon TME

Radiation therapy has been shown to exert beneficial regulatory effects on anticancer immune responses, which include improving antigen presentation, inducing immunogenic cell death, and activating cytotoxic T cells. On the other hand, radiation therapy also has some negative effects on TME. As has been noted, HPD might be related to the previous radiotherapy, for it was often seen in an irradiated field (16, 26, 33, 34, 43). There is plenty of evidence that suggests that radiation treatment can impair the function of immune cells and influence the tumor microenvironment in some ways. First, radiotherapy can promote tumor growth *via* increased vascular endothelial growth factor stimulus and tumor angiogenesis (44), which may accelerate tumor growth. Second, radiation treatment might increase transforming growth factor-β and consequently decrease T cell and dendritic cell activation (45).

Third, radiation-induced secretion of IFN-I may stimulate intrinsic PD-L1 expression in tumor cells and then promote proliferation and radio-resistance of tumor cells (46). However, whether intrinsic PD-L1 expression in tumor cells could mediate HPD occurrence is unclear.

### Upregulation of Alternative Immune Checkpoints

In addition, an upregulation of alternative immune checkpoints stimulated by PD-1 inhibition may lead to tumor immune escape and accelerate tumor growth (47). A typical example might be T cell immunoglobulin mucin-3 expression, which was upregulated in PD-1 antibody bound T cells in tumors progressing following response to anti-PD-1 treatment (47). However, as noted above, there are no signs of HPD in the PACIFIC trial, where all patients were irradiated before durvalumab or placebo in Stage III NSCLC (35). Thus, the relationship between radiotherapy and HPD should be further studied. Further research should be conducted to better clarify the potential link between radiotherapy and HPD.

### ADCP Mediated T Cell Exhaustion

The interaction of the Fc domain of many therapeutic antitumor immunoglobulin Gs (IgGs) with Fcγ receptors (FcγR) has been found to be crucial for their therapeutic activities, which results from the induction of tumor cytotoxicity (48). PD-1 mAb can combine the T cells through the Fab domain while recruiting NK and macrophases through the Fc domain. Thus, NK and macrophages may kill T cells by antibody-dependent cell-mediated cytotoxicity (ADCC) or antibody-dependent cellular phagocytosis (ADCP), which might exhaust T cells and may lead to a rapid tumor progression (48). In an *in vivo* study by Dahan et al. (49), knocking down FcγR of mice augmented the antitumor effect of PD-1 mAb. Russo et al.'s study (17) investigated the role of innate immunity in mediating HPD *via* Fc/FcR in patients with NSCLC. In this study, all 104 HPDs demonstrated tumor infiltration by PD-L1+M2 TAM. Such subtypes of macrophages may mediate HPD by exhausting PD-1 mAb and also stimulate the secretion of the inhibitory cytokine IL-10 (17). These results suggest a pivotal role of tumor-associated macrophage reprogramming on HPD induced by ICIs.

### Activation of Protumoral Effect by Tregs and TAM

T regulatory cells are a highly immunosuppressive T cell subset, which plays a critical role in suppressing the antitumor immune response. Another study analyzed the gastric cancer tissue samples before and after anti-PD-1 mAb therapy and showed that the ICIs obviously increased Ki67+ Tregs in HPDs, compared with that in non-HPDs. *In vitro*, PD-1 blockade significantly enhanced the suppressive activity of Tregs. In a mouse xenograft model, genetic knockout or antibody-mediated blockade of PD-1 in Tregs enhanced their proliferation and suppression of antitumor immune responses (50).

These pieces of evidence are also consistent with a study that has reported that developed-exhausted CD4+ T cells and Tregs increasingly enriched, whereas some effector T cells decreased in HPD observed by scRNA-seq analysis (51). Such imbalance of immune cells may potentially account for the occurrence of HPD. From these findings, we can preliminarily judge that such highly suppressive PD-1+ Tregs may be the possible reason for mediating HPD.

### Tumor-Associated Macrophages

Tumor-associated macrophages (TAMs, same as M2 macrophages) also promote cancer cell proliferation, invasion, and metastasis. A study showed that PD-L1 was also expressed on TAMs, and the presence of PD-L1+ macrophages had been associated with poor prognosis and with immunosuppressive function (52). If activated by ICIs, PD-L1+ macrophages might exert a stronger immunosuppressive effect upon tumor cells in TME. Therefore, we can speculate that TAMs promoted HPD might result from either the Fc pathway or the PD-L1 signal. Further study is expected for the role of PD-L1+ macrophages in HPD occurrence.

PD-1 expression on CD8+ T cells and Treg cells negatively modulates protumoral and immunosuppressive functions, respectively. ICIs may induce both reactivation of dysfunctional PD-1+CD8+ T cells and PD-1+ Tregs. A new study further confirmed the potential role of Tregs behind HPD. The ratio of PD-1+CD8+ T cells to PD-1+ Treg cells in the tumor microenvironment may predict the clinical outcome of ICIs (53).

## Management of HPD

Outcomes for patients who experience HPD remain poor. For the purpose of switching to other potentially efficacious treatments, it is essential to discontinue ICI treatment in HPDs. In theory, rapid tumor growth must be based on accelerated cellular cycles. Thus, prompt administration of cell cycle-dependent cytotoxic chemotherapeutics, such as antimetabolites, taxanes, and vincristine, could be considered for those who develop HPD after ICIs. Similarly, small molecular inhibitors or mAb targeting driven genes should also be chosen, as long as the patient is fit enough. Otherwise, as HPD is always present along with deteriorating clinical status, optimal supportive care and symptomatic therapy may also have great importance. In Kanazu et al.'s study (18), several types of treatment, which include high-dose corticosteroid therapy, antibiotic therapy, and drainage, effectively relieved the symptoms induced by HPD.

As noted above, there is still a lack of reliable clinical and biological markers for early recognition of HPD. Some routinely used clinicopathological parameters, such as age and tumor burden, always failed to provide independent predictors of poor prognosis in Cox regression analysis. Therefore, those suspicious HPDs should be guided by more careful and frequent clinical and biological surveillance to recognize signs of tumor progression. Radiological evaluation remains a key point in physicians' decision-making. Increasing the frequency of radiological assessments may be helpful in some clinical settings. Clinicians have to pay more attention when tumor growth kinetic begins to accelerate during treatment. Otherwise, considering that HPDs are often accompanied by clinical deterioration, its early recognition may be useful for HPD evaluation.

## Summary

Immune checkpoint inhibitors can result in an improved quality of life and durable response in some types of cancer. However, approximately 10% of patients experience HPD, which is thought to be associated with poor quality of life and survival. The discrimination of HPD from that of the naturally progressive disease remains a major challenge in clinical practice. To define and quantify the incidence of HPD, parameters have been applied that include TGR, TGK, PD based on RECIST, and TTF. Conventional PD evaluation, based on the RECIST criterion, will be required to analyze tumor burden, regardless of the time index. HPD evaluation, if based on TGR or TGK, needs to assess both tumor burden and time duration. If by TTF < =2 months, the tumor burden factor will be neglected, otherwise, the evaluation of both TGR and TGK involves mathematical formulae. Currently, there are no standardized definitions of HPD, and the methods of TGR or TGK are not yet widely accepted by the academic community. We think that there might not be any unique cutoff value to identify HPD, no matter using TGR, TGK, TTF, or other methods. In other words, the cutoff value defining HPD may be varied and individualized in different cases. The uncertainty of unique definition could bring some confusion toward HPD evaluation. However, we still do believe that HPD is really occurred in clinical practice and is specifically associated with ICI treatment.

Identifying the high-risk patients with HPD after ICIs may have an important significance on routine clinical work and future studies on immunotherapy. At present, there is still a lack of consistent predictors and biomarkers of HPD. Previous irradiation therapy had been found to be associated with HPD in several cases or case series reports but was not confirmed in the recent phase III trial. In addition, the association with HPD between the different types of ICIs, e.g., antiPD1 and antiPD-L1 is still unclear. Moreover, HPD and pseudoprogression should be paid extra attention to, in the clinic.

Several hypotheses have been proposed to elucidate the underlying mechanisms of HPD. Aberrant alterations in oncogene expression and tumor microenvironment may involve in HPD. At present, the immune nature of HPD has never reached a consensus. To our knowledge, the modulation of subpopulations of immune cells may mainly account for such a phenomenon. Notably, PD1+ Tregs and PD-L1+ TAMs mediated suppressive effects on antitumor immunity appear to be the most reasonable explanation for such a phenomenon. A recovery of effector PD-1+CD8+ T cells rather than PD-1+ Tregs by PD-1 blockade is necessary for tumor regression. The assessment of tumor biopsy samples acquired just at the time of rapid tumor growth will be important to explore the underlying mechanisms of HPD. With regard to HPD treatment, timely administration of cell cycle-dependent cytotoxic chemotherapeutics could be considered. Optimal supportive care and symptomatic therapy may also have great importance. Finally, further studies are warranted to explore the diagnosis, fundamental mechanisms, and the best therapeutic strategies against HPD.

## Author Contributions

XL wrote the draft. LQ revised and modified the final manuscript. All authors contributed to the article and approved the submitted version.

## Funding

XL was supported by the Nature Science Foundation of Gansu Province (No. 21JR7RA618).

## Conflict of Interest

The authors declare that the research was conducted in the absence of any commercial or financial relationships that could be construed as a potential conflict of interest. The handling editor XL declared a shared affiliation with the author XL at the time of review.

## Publisher's Note

All claims expressed in this article are solely those of the authors and do not necessarily represent those of their affiliated organizations, or those of the publisher, the editors and the reviewers. Any product that may be evaluated in this article, or claim that may be made by its manufacturer, is not guaranteed or endorsed by the publisher.

## Abbreviations

dMMR: deficient mismatch repair
EGFR: endothelial growth factor receptor
FcγR: Fcγ receptors
HNSCC: Head and neck squamous cell carcinoma
HPD: hyperprogressive disease
ICI: Immune checkpoint inhibitors
IFN-γ: interferons-γ
IgG: immunoglobulin G
mAb: monoclonal antibody
MDM2/4: murine double minute homolog 2/4
NSCLC: non-small cell lung cancer
OS: overall survival
PD: progressive disease
PD-1: programmed death-1
PD-L1: programmed death ligand-1
PFS: progression-free survival
RECIST 1.1: response evaluation criteria in solid tumors
SLD: sum of the longest diameters
TGK: tumor growth kinetics
TGR: tumor growth rate
TTF: time-to-treatment failure.
